# The ABO Blood Group is an Independent Prognostic Factor in Patients with Ovarian Cancer

**DOI:** 10.7150/jca.36236

**Published:** 2019-10-22

**Authors:** Qian Song, Jun-zhou Wu, Sheng Wang, Zhong-bo Chen

**Affiliations:** 1 Department of Clinical Laboratory, Institute of cancer research and basic medical sciences of Chinese Academy of Sciences, Cancer hospital of University of Chinese Academy of Sciences, Zhejiang Cancer Hospital, Hangzhou, Zhejiang, People's Republic of China; 2 Cancer Research Institute, Institute of cancer research and basic medical sciences of Chinese Academy of Sciences, Cancer hospital of University of Chinese Academy of Sciences, Zhejiang Cancer Hospital & Key Laboratory Diagnosis and Treatment Technology on Thoracic Oncology of Zhejiang Province, Hangzhou, Zhejiang, People's Republic of China; 3 Department of Gynecologic Oncology, Institute of cancer research and basic medical sciences of Chinese Academy of Sciences, Cancer hospital of University of Chinese Academy of Sciences, Zhejiang Cancer Hospital, Hangzhou, Zhejiang, People's Republic of China

**Keywords:** ABO blood group, B antigen, ovarian cancer, prognosis

## Abstract

Previous studies have suggested a relationship between ABO blood group and clinical outcome of various cancers. Nevertheless, little is known about the association between ABO blood group and survival in patients with ovarian carcinoma. This study aimed to investigate the prognostic significance of ABO blood group in patients with ovarian carcinoma. 941 patients who were newly diagnosed with ovarian carcinoma between February 2007 and February 2016 were enrolled in the present study. The relationship between ABO blood type and clinical features in patients with ovarian cancer was analyzed using chi-square tests. Overall survival (OS) stratified by B antigen was evaluated using log-rank test and Kaplan-Meier method. Presence of the B antigen (B/AB) had a worse OS than those in the absence of the B antigen (A/O) in all patients with ovarian cancer, especially in patients with FIGO stage I, IV, and menopause. Presence of the B antigen (B/AB) was significantly correlated with OS than those with non-B antigen (A/O) (hazard ratios 1.342; 95% confidence interval 1.069-1.685; P=0.011). Multivariate analyses revealed that presence of the B antigen (B/AB) was independently associated with OS (hazard ratios 1.532; 95% confidence interval 1.111-2.112; P=0.009). This study indicated that presence of the B antigen (B/AB) was an unfavorable prognostic factor in ovarian carcinoma, especially in patients with FIGO stage I, IV, and menopause.

## Introduction

Ovarian cancer is identified as the seventh most commonly diagnosed carcinoma in the world and the fifth leading cause of carcinoma-related death in women 1-2. Because of the lack of screening for diagnosis of ovarian cancer at early stage, approximately 85 percent of cases of ovarian cancer are diagnosed at an advanced stage (III/IV) 3. Patients with ovarian cancer face with worse prognosis that the 5-year survival rate is only 30% 4. Several factors are correlated with the survival of ovarian cancer, including FIGO stage, residual disease, and grade of differentiation 5-7. Nevertheless, patients with the similar FIGO stage and grade have different prognosis. Therefore, it is imperative to identify accurate, inexpensive and reproducible prognosis factors of ovarian cancer.

Some studies have suggested the relationship between the ABO blood type and the risk of various carcinomas, including colorectal cancer, renal cell carcinoma, and pancreatic carcinoma 8-10. Meanwhile, in a recent analysis of 49,153 women in 1 prospective study, individuals with presence of the B antigen (B/AB) had an increased incidence of ovarian cancer compared with those with non-B antigen (A/O) 11. While several retrospective studies have showed that blood type A was correlated with a higher incidence of ovarian carcinoma compared with blood type O 12-14. However, few studies have evaluated the association between ABO blood group and the prognosis among patients with ovarian cancer. Two retrospective researches reported that blood type A was correlated with a worse clinical outcome in 256 patients with ovarian cancer and in 92 patients with ovarian cancer 15-16. Therefore, we aimed to evaluate the relationship between ABO blood group and the survival of patients with ovarian carcinoma.

## Materials and Methods

### Patient selection

941 patients who were newly diagnosed with primary ovarian carcinoma from February 2007 to February 2016 at the Zhejiang Cancer Hospital were selected for this retrospective research. The diagnosis of ovarian carcinoma was confirmed by a postoperative pathology diagnosis. The criteria of pathology diagnosis were in accordance with the World Health Organization classification standard. The inclusive criteria as follows: (1) ovarian cancer was pathologically confirmed; (2) complete clinical records, including age, FIGO stage, histologic differentiation, menopause, family history of cancer, ascites at surgery, residual disease, and follow-up data; (3) sufficient clinical examination, including ABO blood group, preoperative serum CA125 level. The ABO blood type (A/B/AB/O) was checked by anti-A and anti-B blood grouping reagents (monoclonal antibody). CA125 antigen was quantified in serum in Zhejiang cancer hospital using chemiluminescence microparticle immune assay (CMIA) [ARCHITECT CA125 II Reagent Kit, Abbott]. In the first half year undergoing surgery, patients were followed up at the first, third and sixth months. Patients were followed up every six months during the following years. The follow up examinations include physical examination, serum CA125 level and blood routine examination el at. This retrospective study was approved by the Ethics Committee of Zhejiang Cancer Hospital. All patients provided written informed consent.

### Statistical analysis

The relationship between ABO blood type and clinical features in patients with ovarian carcinoma was analyzed using chi-square tests. The relationship between presence of B antigen (B/AB) and clinical features in patients with ovarian cancer was also evaluated using chi-square tests. Overall survival (OS) was calculated using the Kaplan-Meier method and the log-rank test. Hazard ratio and 95% confidence interval were calculated using COX regression analyses. The overall survival curve was evaluated by GraphPad Prism 7 software. All data were examined by the SPSS software (version 19.0). A value of P < 0.05 was regarded as statistical significance.

## Results

### Patient characteristics

We enrolled 941 patients with ovarian cancer in the study from February 2007 to February 2016. The median age of all individuals was 54 years (range: 23-81 years). 51 patients had FIGO stage I, 76 patients had FIGO stage II, 651 patients had FIGO stage III, and 163 patients had FIGO stage IV. A total of 795 patients had serous cancer, and 146 patients had other. 322 patients had blood type A (34.2%), 250 patients had blood type B (26.6%), 75 patients had blood type AB (8.0%), and 294 patients had blood type O (31.2%). No significant association was determined between the ABO blood group and age, menopause, FIGO stage, family history of cancer, ascites at surgery, residual disease, histology, grade, lymph node stats, Rh factor, and CA125 at diagnosis (Table 1).

### Correlation between presence of the B antigen (B/AB) and clinical features

The correlation between presence of the B antigen (B/AB) and clinical characteristics was listed in Table 2. 325 patients had presence of the B antigen (B/AB) (34.5%), and 616 patients had presence of the non-B antigen (A/O) (65.5%). None of the clinical features was significantly correlated with presence of the B antigen (B/AB), including age, menopause, FIGO stage, family history of cancer, ascites at surgery, residual disease, histology, lymph node stats, Rh factor, and CA125 at diagnosis. Nevertheless, presence of the B antigen (B/AB) was notably associated with grade (P=0.027).

### Prognostic variables for OS

**Figure 1:**
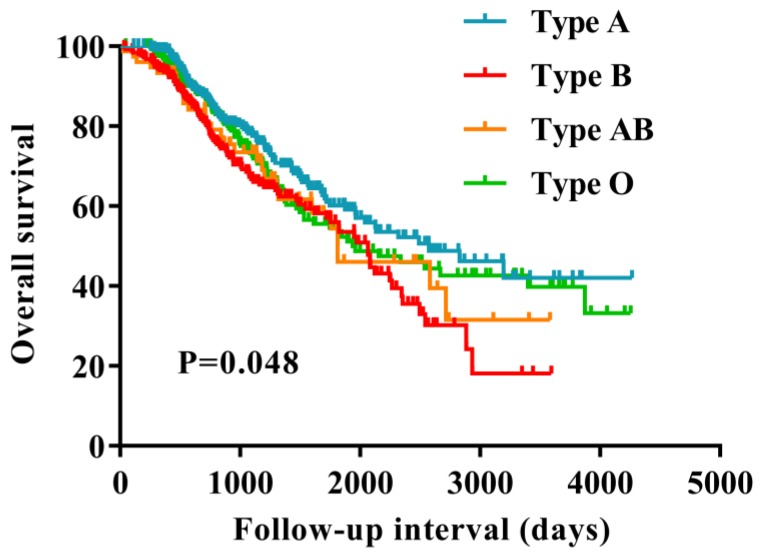
Overall survival for patients with ovarian cancer based on ABO blood type. The patients in blood type B showed significantly worse survival compared to the patients in the non-B blood types (P = 0.048).

**Figure 2:**
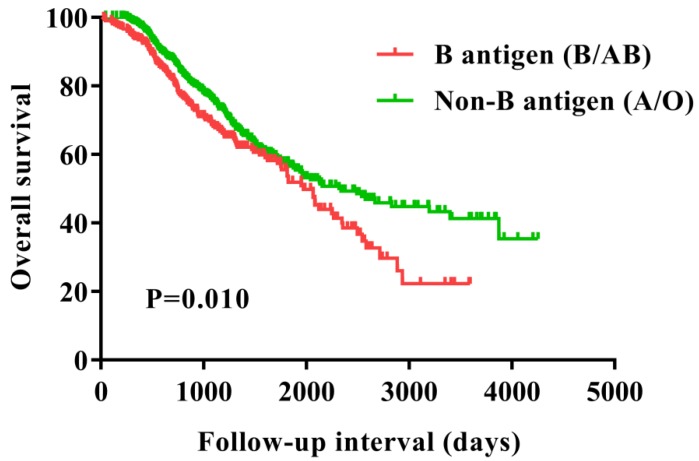
Overall survival for patients with ovarian cancer with B antigen (B/AB) and Non-B antigen (A/O).

The Kaplan-Meier curves suggested that patients in blood type B had a worse survival compared with those in the non-B blood types (P=0.048) (Figure 1). Furthermore, we evaluated the prognostic value of the ABO blood group with presence of the B antigen (B/AB) and non-B antigen (A/O), patients with presence of the B antigen (B/AB) had a worse survival compared to the patients with presence of the non-B antigen (A/O) (P=0.010) (Figure 2).

Univariate analysis indicated that in addition to presence of the B antigen (B/AB) (P=0.011), menopause (P=0.028), FIGO stage I (P=0.008), FIGO stage II (P=0.005), ascites at surgery (P=0.006), residual disease (P=0.008), and lymph node stats (P=0.022) were also significantly correlated with OS. In the multivariate analysis, menopause (P=0.010), FIGO stage I (P=0.008), FIGO stage II (P=0.027), presence of the B antigen (B/AB) (P=0.009) were significantly associated with OS (Table 3).

### Subgroup analysis according to FIGO stage and menopause

**Figure 3:**
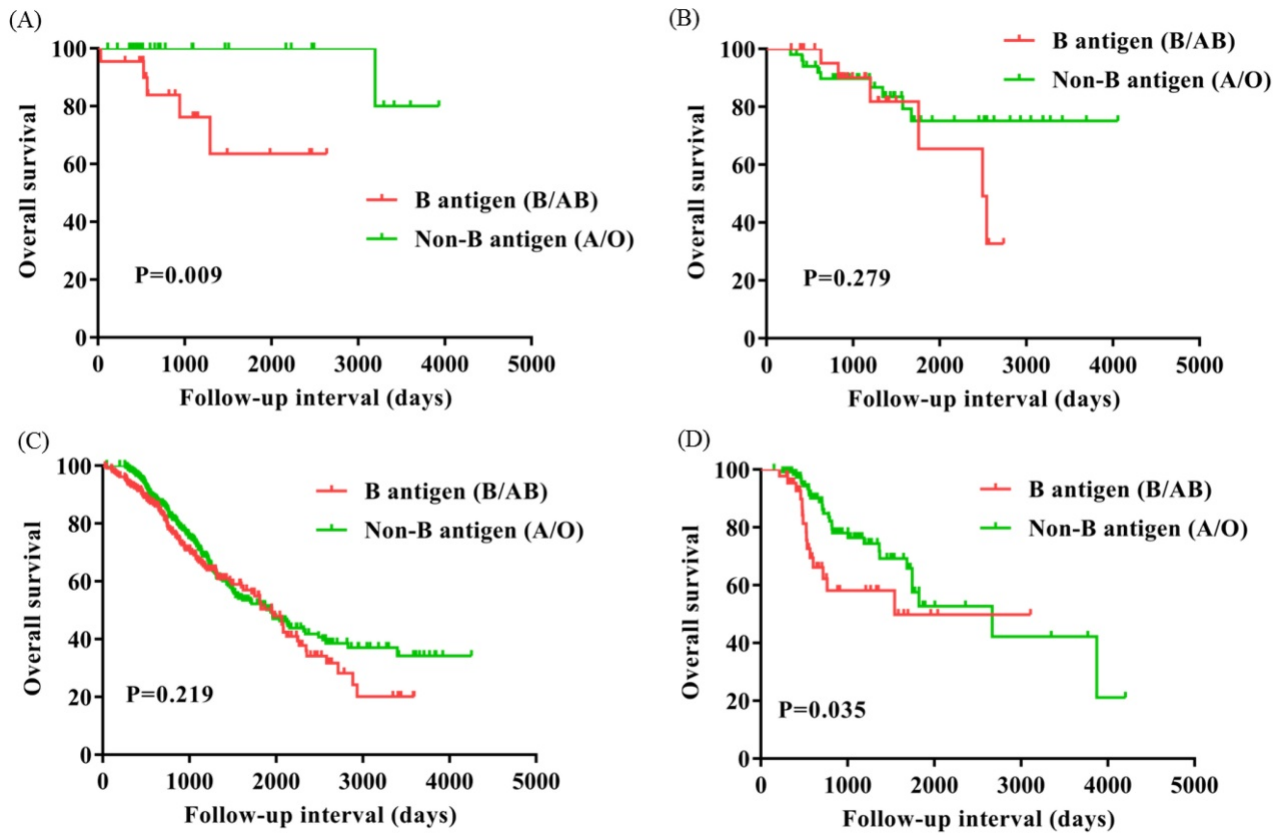
Overall survival for patients with ovarian cancer with B antigen (B/AB) and Non-B antigen (A/O) in patients with FIGO stage I (A), II (B), III (C), and IV (D).

**Figure 4:**
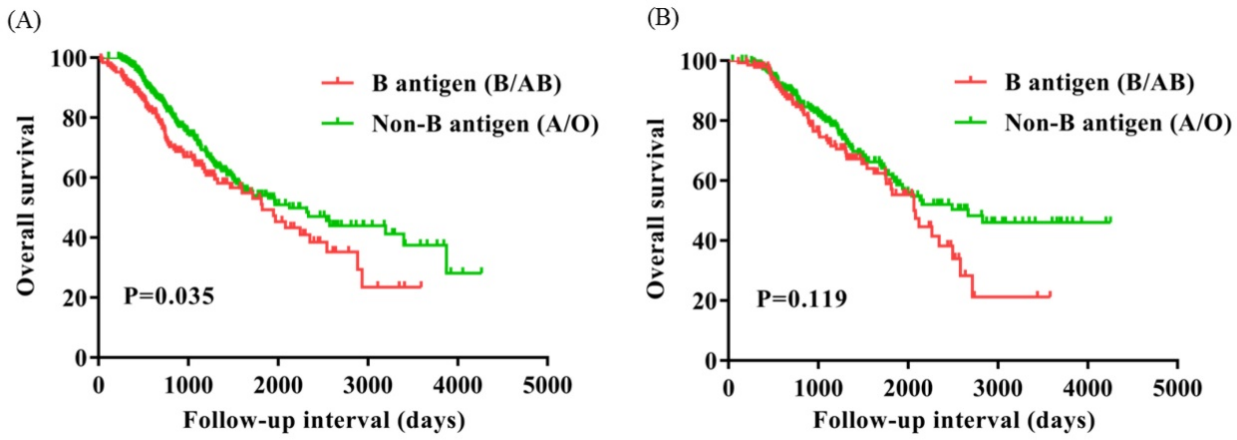
Overall survival for patients with ovarian cancer with B antigen (B/AB) and Non-B antigen (A/O) in patients with menopause (A) and Non-menopause (B).

To evaluate the subgroups of ovarian cancer affected by presence of the B antigen (B/AB), we classified patients based on FIGO stage (I, n=51; II, n=76; III, n=651; IV, n=163) and menopause (Yes, n=542; No, n=399). OS of FIGO stage I and IV were significantly worse for patients with presence of the B antigen (B/AB) (P=0.009 and P=0.035), but OS did not differ neither FIGO stage II nor III (P=0.279 and P=0.219) (Figure 3). OS of patients with menopause was notably worse for patients with presence of the B antigen (B/AB) (P=0.035), but OS of patients without menopause did not differ (P=0.119) (Figure 4).

## Discussions

In this large, retrospective study, blood groups B and AB were significantly associated with worse survival of ovarian cancer. The magnitude of the relationship was similar for blood group B and AB indicating that the B antigen may affect ovarian progression. In analyses of presence of the B antigen (B/AB) compared with absence of the B antigen (A/O), we observed a significantly worse survival in ovarian cancer with FIGO stage I, IV, and menopause.

Previous studies have suggested that the ABO blood group play an important role in the development of various cancers. As ABO antigens are expressed on the surface of several human tissues and cells, such as the ovary surface epithelial cells and ovarian inclusion cysts 17-18. The relationship between the ABO blood group and the cancer risk has been intensely investigated across many different types of cancer, including pancreatic carcinoma, nasopharyngeal cancer, gastric carcinoma, lung carcinoma 19-22. Besides, in a large, prospective study of women, individuals with presence of the B antigen (B/AB) were associated with increased risk of ovarian cancer 11. However, previous retrospective studies have suggested that blood type A had an increased incidence of ovarian cancer 12-14. Proposed reasons for these inconsistent findings were that participants in all studies were from different races, most retrospective reports did not adjust for other possible confounders, and several studies used hospital-based control individual, which may not represent the ABO distribution in the general population 12-13.

There are also many studies have suggested a possible association between the ABO blood group and the clinical outcome in patients with malignant cancers. In 900 patients who underwent resection for renal cell carcinoma, the authors revealed that the non-O blood type was significantly associated with decreased OS 23. Previous study about 404 patients undergoing resection for esophageal carcinoma, there was no relationship between the ABO blood type and the prognosis of esophageal cancer 24. One study showed that blood group A and AB had a shorter OS than others in 333 patients undergoing resection for non-small cell lung cancer 25. Meanwhile, two retrospective reports with a relatively small number of individuals enrolled, regarding the association between the ABO blood group and the clinical outcome of patients with ovarian cancer. Their findings suggested that a negative relationship between blood type A and ovarian cancer survival, but no relationship with blood type B 15-16. Owing to these results, we hypothesized that whether blood type A or blood type B might associate with prognosis of ovarian cancer. We found that blood group B and AB indicated worse survival in patients with ovarian cancer, especially in patients with FIGO I, IV, and menopause. The reasons for these conflicting results were that a relatively large number of participants enrolled without exclusion of FIGO stage IV, the proportions of ABO blood group were different. 256 patients with ovarian cancer in previous study, 60 patients had blood group A (23.4%), 52 patients had blood group B (20.3%), 24 patients had blood group AB (9.4%), and 120 patients had blood type O (46.9%) 15. 941 patients enrolled in our study, the ABO distribution was 322 patients with blood type A (34.2%), 250 patients with blood type B (26.6%), 75 patients with blood type AB (8.0%), and 294 patients with blood type O (31.2%).

The mechanisms of the ABO blood group and the survival of cancer have not been intensely investigated. As A and B antigens are also expressed on the ovarian cancer tissues 17-18. We hypothesized that the ABO antigens in cancer cells contribute to the pathway signaling, intracellular adhesion, and inflammation, all of which play an important part in the progression of carcinoma 26. The expression of ABO blood antigens on tumor cells was influenced by hyper-methylation of ABO promoter, which was associated with the tumor progression. ABO promoter hyper-methylation was also checked in dysplastic and hyperplastic tissues adjacent to tumor, indicating that it was an early event in the development of carcinoma 27. Therefore, B antigen was correlated with the survival of patients in the FIGO stage I. The ABO gene, which is located on chromosome 9q34, encodes glycosyltransferases to form the ABO blood group antigens 28. The ABO gene is one frequent region of deletion in ovarian cancer 29. Aberrant glycosylation was one of the most important hallmarks of tumor 30. Therefore, we speculate that patients with presence of B antigen (B/AB) have more malignant cancer cells. Patients in the FIGO stage IV showed the worse survival.

There are several limitations of this analysis: first, this study was a retrospective and single-center analysis. Second, the clinical results regarding the ABO blood types were determined by a serological technique according to the phenotype, not the genotype. Third, all the participants of this analysis were Chinese, perhaps restricting the generalizability of these findings. Therefore, further investigations are required to clarify the reproducibility of this study, including the multi-center analysis and the investigation of different races from other countries.

In summary, our results show that there are a possible association between the B blood group antigen and worse clinical outcome of ovarian cancer. Patients with presence of the B antigen (B/AB) had a significantly worse survival than those with non-B antigen (A/O), especially in patients with FIGO stage I, IV, and menopause. However, given the inconsistent findings of previous studies, additional investigations are urgently needed to evaluate this association. Researches of potential mechanisms for a relationship between the ABO blood group and ovarian cancer prognosis also are needed.

## Figures and Tables

**Table 1:** Relationship between ABO blood group and clinicopathological features in patients with ovarian cancer

Characteristics	Total (N=941)	A (N=322)	B (N=250)	AB (N=75)	O (N=294)	*P* value
**Age[median (range),years]**	54 (23-81)	55 (23-80)	54 (25-80)	55 (36-77)	53 (26-81)	
≤55	503	170	134	38	161	0.920
>55	438	152	116	37	133	
**Menopause**						
Yes	542	191	142	47	162	0.574
No	399	191	108	28	132	
**FIGO stage**						
I	51	16	17	5	13	0.504
II	76	25	18	7	26	
III	651	218	184	50	199	
IV	163	63	31	13	56	
**Family history of cancer**						
Yes	307	104	76	29	98	0.595
No	634	218	174	46	196	
**Ascites at surgery**						
Yes	448	164	108	31	145	0.523
No	171	63	43	17	48	
Unkown	322					
**Residual disease**						
≤1cm	817	281	215	64	257	0.932
>1cm	124	41	35	11	37	
**Histology**						
Serous	795	275	206	65	249	0.725
Other	146	47	44	10	45	
**Grade**						
Well	363	133	84	26	120	0.286
Moderate	223	68	69	22	64	
Poorly	253	89	63	18	83	
Unkown	102					
**Lymph node stats**						
Positive	532	187	139	38	168	0.683
Negative	409	135	111	37	126	
**Rh factor**						
Positive	937	319	250	74	294	0.125
Negative	4	3	0	1	0	
**CA125 at diagnosis**						
≤35 U/ml	55	19	12	5	19	0.851
>35 U/ml	886	303	238	70	275	

FIGO, International Federation of Gynecology and Obstetrics.

**Table 2:** Relationship between B antigen and clinicopathological features in patients with ovarian cancer

Characteristics	Non-B antigen (A/O) (N=616)	B antigen (B/AB) (N=325)	*P* value
**Age[median (range),years]**	54 (23-81)	55 (25-80)	
≤55	331	172	0.813
>55	285	153	
**Menopause**			
Yes	353	189	0.802
No	263	136	
**FIGO stage**			
I	29	22	0.092
II	51	25	
III	417	234	
IV	119	44	
**Family history of cancer**			
Yes	202	105	0.880
No	414	220	
**Ascites at surgery**			
Yes	309	139	0.333
No	111	60	
Unkown			
**Residual disease**			
≤1cm	538	279	0.520
>1cm	78	46	
**Histology**			
Serous	524	271	0.498
Other	92	54	
**Grade**			
Well	253	110	**0.027**
Moderate	132	91	
Poorly	172	81	
Unkown			
**Lymph node stats**			
Positive	355	177	0.351
Negative	261	148	
**Rh factor**			
Positive	613	324	0.688
Negative	3	1	
**CA125 at diagnosis**			
≤35 U/ml	38	17	0.560
>35 U/ml	578	308	

FIGO, International Federation of Gynecology and Obstetrics.

**Table 3:** Overall survival analyses according to ABO blood group in patients with ovarian cancer

Variables	Univariate			Multivariate		
	HR	95% CI	*P* value	HR	95% CI	*P* value
**Age ( >55 vs. ≤55)**	1.200	0.960-1.499	0.109			
**Menopause (No vs. Yes)**	1.288	1.027-1.615	**0.028**	1.530	1.105-2.119	**0.010**
**FIGO stage**						
I	0.317	0.135-0.744	**0.008**	0.297	0.121-0.727	**0.008**
II	0.430	0.239-0.775	**0.005**	0.476	0.247-0.919	**0.027**
III	1.099	0.796-1.516	0.567	1.121	0.778-1.615	0.541
IV	1.000					
**Family history of cancer (No vs. Yes)**	0.845	0.663-1.077	0.173			
**Ascites at surgery (No vs. Yes)**	1.706	1.166-2.497	**0.006**	1.461	0.981-2.175	0.062
**Residual disease (>1cm vs. ≤1cm)**	1.454	1.102-1.918	**0.008**	1.163	0.735-1.841	0.519
**Histology (Other vs. Serous)**	1.113	0.826-1.500	0.481			
**Grade**						
Well	0.830	0.583-1.182	0.302			
Moderate	1.119	0.862-1.453	0.400			
Poorly	1.000					
**Lymph node stats (Negative vs. Positive)**	1.303	1.038-1.635	**0.022**	0.924	0.656-1.301	0.652
**CA125 at diagnosis (>35 U/ml vs. ≤35 U/ml)**	1.144	0.669-1.955	0.624			
**Blood type**						
A	0.844	0.633-1.126	0.249			
B	1.258	0.947-1.671	0.112			
AB	1.147	0.752-1.749	0.524			
O	1.000					
**A antigen [Absent (O/B) vs.Present (A/AB) ]**	0.807	0.642-1.015	0.067			
**B antigen [Absent (O/A) vs. Present (B/AB)]**	1.342	1.069-1.685	**0.011**	1.532	1.111-2.112	**0.009**
